# Exploring Current Trends, Challenges and Future Directions of Intraoral Digital Impression in the Management of Patients with Cleft Lip and/or Palate: A Narrative Literature Review

**DOI:** 10.3390/children12121579

**Published:** 2025-11-21

**Authors:** Jyotsna Unnikrishnan, Mahmoud Bakr, Robert M. Love, Ghassan Idris

**Affiliations:** 1 School of Medicine and Dentistry, Griffith University, Gold Coast, QLD 4222, Australia; m.bakr@griffith.edu.au (M.B.); r.love@griffith.edu.au (R.M.L.); g.idris@griffith.edu.au (G.I.); 2 Children’s Oral Health Service and Child Specialist Services, Metro North Hospital and Health Services, Queensland Children’s Hospital, South Brisbane, QLD 4101, Australia

**Keywords:** cleft lip, cleft palate, clinical challenges, dental impression technique, dental, infant, intraoral scanners, neonatal screening

## Abstract

**Introduction**: Cleft lip and palate (CL/P) patients require long-term interdisciplinary care to enhance function, aesthetics, and quality of life. Digital impressions (DI) using intraoral scanners (IOS) have become a viable substitute for traditional impressions in all areas of dentistry, including CL/P care. This review summarises the literature on DI’s potential to replace conventional impressions (CI) in the care of CL/P patients, evaluating clinical integration, accuracy, patient and clinician perceptions, and implementation challenges. **Methods**: A comprehensive literature search was performed across PubMed, Scopus, Web of Science, Embase, Cochrane Library, and Google Scholar to identify all published studies utilising digital impressions in the clinical care of cleft lip and palate (CL/P) patients up to March 2024. Predefined inclusion and exclusion criteria were applied. Out of 503 initially retrieved records, 27 studies met the final eligibility criteria and were included in this review. **Results**: DI demonstrated comparable accuracy to CI in capturing oral structures in CL/P patients, with minimal discrepancies in intra-arch measurements. Patients and parents perceived DI as less invasive and more comfortable, while clinicians noted reduced respiratory complications. Challenges included capturing deep cleft areas and managing unique neonatal and infant anatomy. The review highlights the need for further research on optimal scanning techniques, scanner design, and standardised protocols to enhance DI effectiveness in CL/P care. **Conclusions**: DI is a viable alternative to CI in CL/P management, offering efficient, patient-centred workflows. Integrating digital technologies can enhance clinical outcomes, but ongoing research is essential to refine scanning strategies, improve accuracy, and address anatomical challenges in this population.

## 1. Introduction

Cleft lip and palate (CL/P) is a prevalent congenital craniofacial anomaly, occurring at a global rate of roughly 7.94 per 10,000 live births, with variations ranging from 3.13 in South Africa to 19.2 in Japan [1]. Children born with CL/P require long-term, multidisciplinary management commencing at birth, with accurate documentation being essential for effective treatment planning and outcome evaluation [2]. According to the World Health Organisation (WHO), study models should be recorded soon after birth, before lip repair, between 5 and 10 years of age, and again between 18 and 20 years [3].

Orthodontic intervention, often referred to as early maxillary orthopaedics or pre-surgical orthopaedic treatment (PSOT), forms an integral part of the comprehensive management of CL/P [4,5]. The initial oral impression taken in infancy serves as both a diagnostic aid and a means of fabricating pre-surgical infant orthopaedic (PSIO) appliances. Repeated impressions at different stages of treatment allow clinicians to evaluate growth and the effectiveness of interventions [3]. The accuracy of both the impression and the fabricated appliance play a pivotal role in determining the quality of the surgical outcome. Hence, ensuring precision and reproducibility during the scanning process is essential for clinical success [5]. Recent advancements in digital dental technology, particularly intraoral digital impressions (DI) and digital models, are beginning to transform the treatment approach for this vulnerable population [6,7,8]. However, conventional impression (CI) techniques using alginate or rubber-based materials are highly technique-sensitive and pose considerable risks for neonates, particularly airway obstruction and cyanotic episodes [9]. These risks arise from factors such as the engagement of undercuts, material fragmentation, and backflow into the oropharynx, all of which can lead to respiratory compromise. As infants are obligatory nasal breathers, even minor obstructions can result in significant oxygen desaturation during the procedure [10]. Restricted mouth opening, limited visibility, and the mobility of the premaxilla further complicate the accurate recording of cleft morphology [11,12].

To minimise complications, impressions in neonates with clefts should be performed in hospital settings equipped to manage airway emergencies [5]. The infant must remain awake during the procedure, and crying is considered a reassuring sign of an open airway. High-volume suction should be available to manage regurgitation, and custom trays are recommended to capture the complete maxillary anatomy, though these add complexity and preparation time [13]. Despite these precautions, the conventional approach remains challenging and carries inherent risks.

DI offers several advantages over traditional methods, including enhanced accuracy, reduced chair time, and improved patient comfort. The elimination of impression materials significantly decreases the risk of airway obstruction, making this technology especially beneficial for paediatric and cleft patients. Moreover, digital models overcome the limitations of plaster casts, such as the need for physical storage, transportation difficulties, and the risk of damage or loss [14].

Nevertheless, integrating DI into CL/P management is not without challenges. Anatomical variations associated with cleft conditions may complicate the scanning process, and the cost of intraoral scanning technology can limit access, particularly in resource-constrained settings. Additionally, clinicians require adequate training to effectively utilise digital tools and interpret the resulting data, underscoring the need for educational updates and workflow adaptation.

The management of CL/P represents a complex intersection of surgical, orthodontic, and prosthodontic disciplines. The emergence of DI technologies holds great potential to enhance diagnostic precision, treatment efficiency, and overall patient safety. This narrative review aims to critically evaluate the current evidence on the use of DI in the management of patients with CL/P. Specifically, it examines the potential of DI techniques to replace or complement conventional impression methods, assessing their clinical accuracy, safety, efficiency, and user acceptability among both patients and clinicians. Furthermore, the review explores the extent of integration of digital technologies into multidisciplinary CL/P care, identifies barriers to their widespread adoption, and highlights gaps in current knowledge to guide future research and clinical innovation.

## 2. Materials and Methods

A comprehensive search strategy was employed to identify studies related to the use of DI and IOS in the treatment of patients with CL/P. The electronic search was conducted across six databases—PubMed, Scopus, Web of Science, Embase, Cochrane Library, and Google Scholar—using keywords including cleft, lip, palate, orofacial, alveolus, intraoral scans, 3D scans, digital models, and digital workflow. Search terms were combined using Boolean operators [AND/OR] and applied consistently across all databases to maximise the capture of relevant literature addressing digital applications in CL/P diagnosis, treatment, and workflow integration (Table 1). To ensure completeness, electronic searches were supplemented by manual searches of key orthodontic and craniofacial journals, including American Journal of Orthodontics and Dentofacial Orthopaedics, European Journal of Orthodontics, Angle Orthodontist, and The Cleft Palate–Craniofacial Journal. Reference lists of all included studies were also reviewed to identify any additional relevant articles.

No restrictions were applied regarding the year of publication, as the aim was to capture both historical developments and the most recent advancements in digital approaches to CL/P management. Similarly, no limitations were placed on patient age or gender. The literature search was conducted up to March 2024, ensuring inclusion of the most recent and relevant studies available at the time of review.

Inclusion criteria comprised studies that discussed the application of digital technologies in CL/P treatment, specifically those using IOS to produce digital models. Exclusion criteria included studies using digital models generated from laboratory scanners, those involving cone-beam computed tomography (CBCT), non-English publications, conference abstracts, and studies not specifically addressing CL/P treatment.

All retrieved records were imported into Covidence for systematic management and screening, with duplicates automatically removed. Two independent reviewers screened the titles and abstracts of the identified studies, followed by full-text evaluation according to the predefined inclusion and exclusion criteria. Extracted information was organised under key themes, including current applications of digital technologies in CL/P management, accuracy, patient- and clinician-related outcomes, identified challenges and limitations, and proposed future directions. The findings from individual studies were compared and summarised to highlight emerging trends, gaps in evidence, and areas for future research. Any disagreements between reviewers were resolved through discussion and consensus, and when necessary, a third reviewer was consulted to reach a final decision. As this study followed a narrative review approach, data were synthesised qualitatively to summarise key themes rather than through a systematic or quantitative process.

**Figure 1:**
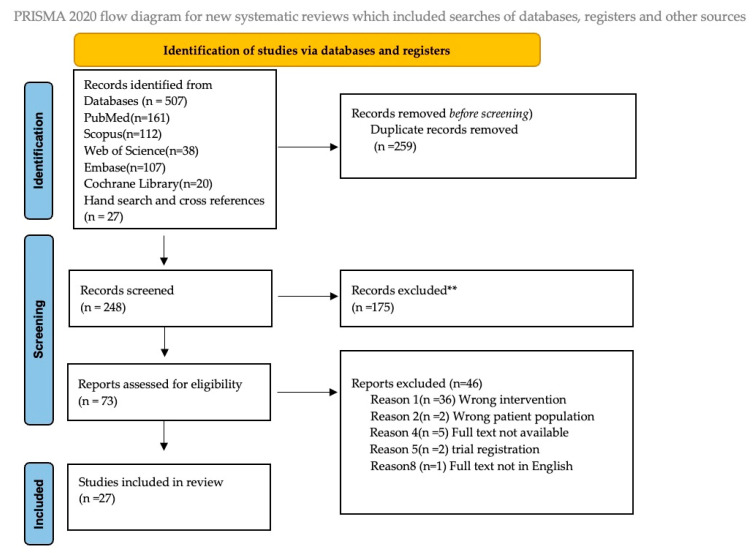
PRISMA Flow chart-The study selection process.

A total of 503 articles were initially identified. After the removal of duplicates and screening of titles, abstracts, and full texts, 27 studies met the inclusion criteria and were included in the final review. The study selection process is summarised in the PRISMA flowchart Figure 1.

## 3. Results

A total of 27 studies that met the inclusion criteria were analysed and are summarised in Table 1. These studies explored the use of DI obtained through IOS or direct digital models (DDM) in the management of patients with cleft lip and palate (CL/P). The selected studies were published between 2016 and 2024, reflecting the growing integration of digital technologies into clinical and research practices related to cleft care.

The included studies were categorised based on their primary focus areas: accuracy, patient comfort, time efficiency, use as diagnostic aids, treatment planning or treatment monitoring, and use as outcome measures. Among the 27 studies, 7 primarily evaluated the accuracy of DI or digital models when compared with conventional techniques. Five studies examined patient comfort, reporting improved acceptance and reduced discomfort associated with intraoral scanning compared with traditional impressions. Five studies assessed the time required, generally indicating shorter clinical chairside time and improved workflow efficiency.

In addition, four studies investigated the use of DI as diagnostic aids, highlighting their value in visualising complex cleft morphologies and facilitating interdisciplinary communication. Ten studies focused on treatment planning and treatment evaluation, demonstrating the potential of digital models to assist in surgical and orthodontic planning, simulate treatment outcomes, and enhance precision in interdisciplinary management. Finally, four studies utilised direct digital models as outcome measures, allowing objective evaluation of treatment results and longitudinal comparison of morphological changes (Table 2).

## 4. Discussion

### 4.1. Current Trends in the Use of Intraoral Scanning in Patients with CL/P: Accuracy, Feasibility, and Clinical Application

#### 4.1.1. Accuracy and Reliability of DI in CL/P Patients

Accurate reproduction of the complex intraoral anatomy in CL/P patients is essential for diagnosis, appliance fabrication, and treatment planning. Recent research consistently demonstrates that IOS can achieve accuracy comparable to CI, though variations may occur depending on cleft morphology and clinical handling (Table 3).

Patel et al. [12] compared direct digital models obtained using an IOS with conventional plaster models in infants with bilateral CL/P (BCL/P). The greatest surface discrepancy occurred in the premaxillary segment, largely attributed to soft tissue compression during conventional impression making rather than limitations of the digital method. This highlights that DI minimises tissue distortion inherent in CI techniques, thereby improving the fidelity of digital models.

ElNaghy et al. evaluated the precision of DI versus CI in infants with unilateral CL/P (UCL/P) and reported excellent agreement between the two, with dimensional differences ranging from only 0.01 to 0.1 mm [31]. These results confirm that IOS—particularly 3Shape TRIOS systems, which were the most frequently used scanners across studies—can accurately capture fine intraoral details even in irregular cleft morphologies. Other studies using Carestream and Medit i700 (Medit Corp, Seoul, Republic of Korea) scanners also reported no statistically significant differences in model accuracy compared with CI, confirming the reliability of different IOS technologies in CL/P applications [15,17,33,36,37].

Unnikrishnan et al. analysed intra-arch measurements and surface discrepancies between digital models generated from DI and those obtained using conventional alginate and silicone-based impressions. No statistically significant difference (*p* < 0.05) was observed between the superimposed models, reinforcing the accuracy and reproducibility of DI in CL/P patients [37]. However, there are currently no comparative studies evaluating the accuracy of different intraoral scanners in CL/P patients, which highlights an important area for further research.

Zeidan and Kamiloglu et al. [26] extended these findings by comparing indirect digital models obtained from IOS of plaster models with those generated from Cone Beam Computed Tomography (CBCT) scans. Apart from posterior cleft width, all intra-arch measurements demonstrated excellent reliability, underscoring the consistency of IOS in capturing detailed oral structures [26].

Similarly, Dalessandri et al. demonstrated that a DI-based workflow in PSOT for newborns with CL/P showed minimal deviation from the traditional tray and putty method (mean difference: −0.2 mm to −0.3 mm) [7]. A recent systematic review also confirmed that DI in infants with CL/P provides accuracy comparable to CI for intra-arch measurements [39].

#### 4.1.2. Patients’, Parents’ and Clinicians’ Experience with DI in CL/P Patients

##### Patients’ or Parents’ Experience with DI in CL/P Patients

Patient and parent perception plays a vital role in the successful adoption of digital technologies in CL/P management. Across multiple studies, DI using IOS has consistently been perceived as more comfortable, less invasive, and less distressing compared to CI (Table 4).

Chalmer et al. [15] compared comfort and time requirements between CI and DI in infants with CL/P, using structured questionnaires completed by patients’ parents. Scanning comfort was rated substantially higher (84.8%) than CI (44.2%) (*p* < 0.05). Although the perceived scanning time was slightly longer (56.6% vs. 51.2%), this difference was not statistically significant (*p* > 0.05). Importantly, none of the participants reported disliking digital scanning, whereas 16.3% expressed discomfort with CI [15]. These findings highlight the superior patient and caregiver tolerance of DI, particularly for infants who may experience respiratory distress or gagging during CI procedures.

Dalessandri et al. assessed mothers’ perceptions of tray-and-putty impressions versus DI obtained using a 3Shape TRIOS scanner, reporting that DI was viewed as less invasive and easier to tolerate during PSOT [7]. Similar observations were reported by Fomenko et al. and Soliman et al., both of whom found that parents preferred DI due to the absence of impression materials, reduced risk of airway obstruction, and improved overall comfort [17,36].

A recent systematic review further consolidated these findings, showing a clear preference among parents and clinicians for DI in infants with CL/P. The review highlighted additional advantages, including the ability to pause or rescan during the procedure, better visualisation of the oral anatomy, and immediate availability of digital records [39]. While most studies utilised 3Shape TRIOS scanners, other scanning systems have also demonstrated similar patient acceptance and ease of use.

Overall, current evidence suggests that DI provides a safer, more comfortable, and more efficient experience for CL/P patients and their parents compared with CI, supporting the broader clinical shift toward digital workflows in craniofacial care.

##### Clinicians’ Experiences and Practical Challenges with DI in CL/P Patients

Clinical experience within CL/P care demonstrates substantial advantages in safety and workflow, though challenges remain in capturing complex anatomy. Most studies report that DI provides satisfactory clinical outcomes, yet deep cleft regions and undercuts are often difficult to record completely [6,22,33] (Table 4).

Operator expertise and scanner design play crucial roles in accuracy and efficiency. Gong et al. noted that scanners with smaller tips and faster acquisition speeds, such as the *3Shape TRIOS* and *Medit i700*, facilitated easier scanning, though infant uncooperativeness often necessitated rescanning [22]. Shanbhag et al. similarly reported that multiple scans were needed due to movement, with smaller scanner heads preferred for procedures lasting about 20 min [23]. Batra et al. found that “child-sized” scanning tips reduced procedure time to 90–120 s [40].

Wiese et al. observed that scanning deeper palatal clefts required the use of cotton swabs to bridge tissue gaps, extending scan times to a median of 151 s [25]. ElNaghy et al. confirmed similar challenges, with operators using bonding brush handles and reporting scan durations of 80–120 s [31]. Excessive salivation and movement also prolonged scanning in some cases, as described by Okazaki et al. and Soliman et al. [33,36].

Although large cohort studies reported no adverse events, scan time varied by cleft severity, ranging from 60 s for cleft palate to 150 s for bilateral CL/P [8]. Post-processing challenges, such as trimming digital casts to correct incomplete surface capture, were also reported [28].

Overall, current evidence supports DI as a safe and efficient alternative to CI. However, limitations persist in scanning deep clefts and managing infant cooperation. Importantly, no comparative studies have evaluated the performance of different IOS systems, indicating a key direction for future research.

#### 4.1.3. Clinical Application of DI in CL/P Patients

DI obtained through IOS has become an integral part of the clinical management of patients with CL/P. They provide accurate 3D representations of the cleft anatomy, supporting diagnosis, treatment planning, appliance fabrication, and assessment of treatment outcomes across various stages of care (Table 5).

##### Diagnostic Use of DI in CL/P Patients

The diagnostic application of DI has expanded rapidly in recent years. Studies have demonstrated that digital models generated by IOS can accurately reproduce cleft morphology and maxillary arch dimensions in both unilateral (UCL/P) and bilateral (BCL/P) cases [12,16,27,34,41]. Choi et al. used DI to evaluate maxillary arch width and cleft dimensions pre-palatoplasty, confirming its reliability as a non-invasive diagnostic tool. The study also illustrated how 3D-scanned images can be used to create digital cleft models for training surgical residents, enhancing understanding of palatal contours and enabling simulation-based education [16].

Woodsend et al. [27] further demonstrated the diagnostic potential of DI by developing digital models for automated landmark identification in the deciduous dentition using machine learning. This application of digital models enhances diagnostic precision and standardises measurement in CL/P assessment [34,41].

Moreover, some studies have explored the use of IOS for extraoral scanning, integrating nasal and lip morphology into 3D craniofacial analyses [29,42,43,44]. These approaches highlight the potential of DI to complement traditional imaging modalities and provide comprehensive assessments of cleft anatomy.

##### Treatment Planning or Treatment with DI in CL/P Patients

DI obtained through IOS has become an increasingly valuable component in the treatment planning and management of CL/P patients, particularly in the design and fabrication of presurgical nasoalveolar moulding (NAM) appliances. Several studies have reported the successful integration of fully digital workflows for NAM fabrication, involving direct intraoral scanning, computer-aided design (CAD), and additive manufacturing [6,8,18,19,21,23,24,25,30]. This approach reduces reliance on CI, which are often challenging in newborns due to limited oral access and potential airway risks.

Compared to traditional methods, CAD-based NAM fabrication offers notable advantages, including improved precision, reproducibility, and patient comfort. For example, Batra et al. and Meyer et al. described the use of digitally designed sequential NAM aligners incorporating controlled expansion of the posterior arch width to support transverse growth [20,35]. Similarly, Gong et al. demonstrated that digital workflows significantly reduced manual adjustments, improved appliance fit, and decreased chairside time and the number of clinical visits for both clinicians and families [45].

Nonetheless, challenges remain. Accurate intraoral scanning in infants with wide or deep clefts is technically demanding, often requiring multiple scans or specialised auxiliary tools to capture complex undercut areas. Digital trimming and segmentation of models can also be limited when cleft margins are deep or irregular, as current software algorithms may not always accurately delineate these regions.

Overall, the integration of DI and CAD-based workflows has transformed NAM appliance fabrication by enhancing standardisation, customisation, and clinical efficiency. Future studies should aim to evaluate the long-term clinical outcomes, cost-effectiveness, and the potential of integrating newer IOS technologies with automated design systems to further improve precision and workflow efficiency.

##### Outcome Measures with DI in CL/P Patients

IOS has emerged as a valuable tool for assessing treatment outcomes in patients with CL/P, enabling precise evaluation of NAM and surgical procedures through accurate digital measurements [7,15,17,30,32,45,46]. Chalmers et al. [15] evaluated the use of DI as an alternative to CI, applying digital models derived from IOS to assess intra-arch measurements using the GOSLON and MHB indices. These indices served as objective measures for determining surgical outcomes. Similarly, Gong et al. investigated a comprehensive digital workflow for the design and fabrication of NAM appliances, assessing treatment effectiveness by comparing intraoral scanned images captured pre- and post-treatment. The use of IOS-generated digital models facilitated treatment modifications, thereby enhancing clinicians’ ability to monitor progress and improve clinical outcomes.

### 4.2. Challenges Related to DI in Patients with CL/P

Even though DI and 3D printing techniques in the care of patients with CL/P have seen significant advancement in recent years, DI using IOS could pose some challenges in neonates, infants and children with cleft lip and palate that impact effectiveness and efficiency. These challenges influence both the effectiveness and efficiency of digital workflows [39]. One primary difficulty reported across the studies is the capture of deep cleft areas, which often result in incomplete scans. This limitation is primarily attributed to the small mouth opening of the children, the large scanner head and the inability of the scanner head to reach the deepest part of the cleft defect [6,33]. Studies using TRIOS (3Shape, Copenhagen, Denmark) and iTero (Align Technologies, Tempe, AZ, USA) scanners noted that smaller scanner tips and pre-warming the scanner head improved accessibility and patient comfort, although deep clefts remained difficult to image completely.

A distinctive feature in this population is the discontinuity of the dental arch caused by the alveolar cleft. Considerations should be given to bridge the cleft gap before palatal repair, especially when the clefts are deeper and wider. Novel methodologies have been explored to overcome this, as most scanning software algorithms lack the capability to bridge the cleft defect automatically. Weise et al. described ways to build a ‘virtual bridge’ between the cleft segments by either inserting cotton swabs or using the tip of a glove to connect the gaps in the cleft segments during scanning. Similarly, another investigation employed bonding brush handles to bridge the gaps, facilitating more accurate alignment in wider clefts [6,31]. These innovations demonstrate how simple, low-cost adjuncts can improve data capture across different scanner systems. Moreover, an altered scanning pattern that includes intact areas of the lip, jaw, palate, and nose as reference points has been shown to enhance scanning.

Other limitations highlighted include head movement, excessive salivation, and restricted mouth opening, all of which affect image stitching and the accuracy of scans in infants [23,35]. Excessive salivation has been shown to have a negative impact on the quality of scans obtained during DI [32]. To mitigate these challenges, clinicians have suggested utilising a smaller scanner tip, a pre-warmed scanning tip, and the use of scanners with a faster scanning process. The presence of a cleft and these adverse intraoral factors underscores the significance of skilled operators who are proficient in scanning to provide a high-quality scan [47]. Research has demonstrated that the operator’s proficiency substantially impacts the precision of DIs produced by the various intraoral scanning systems (IOS) [48,49].

Cost and accessibility also play a decisive role in the adoption of IOS technology. The financial investment for IOS devices ranges from USD 12,000 to 35,000, with additional annual software subscription costs [50]. Despite this, multiple studies concluded that DI becomes cost-effective over time, particularly in high-volume clinical environments. For example, practices performing two DIs daily could recover costs in approximately one year, while those performing five could do so in five months. Compared with CI, which requires ongoing expenses for trays, alginate, plaster, and trimmers, DI substantially reduce material use and chairside time [50].

Considering the reduced number of plaster models, patient visits, and time required to create individual casts, treatment planning and treatment using digital models in NAM can be cost-effective in CL/P care [7,51,52]. Studies such as those by Grill et al. demonstrated that semi-automated rapid NAM plates fabricated via digital workflows were more economical than conventional NAM plates [53]. However, when the costs of scanners, 3D printers, and software are included, digital workflows may remain challenging for smaller or resource-limited centres.

Despite these limitations, the global adoption of IOS is increasing—an international survey of 1072 respondents across 109 countries found that 78.8% of clinicians use IOS in daily practice, representing 36 scanner types and 38.6% using CAD software, underscoring the rapid integration of digital workflows into modern dentistry [54]. A recent study presented a comprehensive, step-by-step protocol for integrating digital scanning into the presurgical infant orthopedics (PSIO) workflow in cleft lip and palate (CLP) care [55]. Another protocol, successfully implemented across two cleft centres, detailed the optimal positioning of the patient, clinician, scanner, and monitor, enabling accurate digital capture of the lip, nose, and cleft palate within approximately one minute in both outpatient and operative settings [56]. This work provides practical guidance for incorporating intraoral scanning into early cleft management and reflects the growing shift toward adopting digital impressions as a routine practice in CLP care, replacing conventional impression techniques.

### 4.3. Future Pathways in DI for Cleft Care

The ideal scanner for managing cleft lip and palate patients, especially neonates and infants, must overcome current limitations in capturing complex intraoral anatomy. Essential features should include the ability to capture the deep parts of the cleft region accurately, rapid image acquisition to minimise discomfort and a smaller scanner head for improved manoeuvrability within the confined spaces of the oral cavity. Additionally, an advanced software algorithm is essential for accurately capturing the intricate details of a cleft, where arch discontinuities are present.

Hence, the factors that need to be explored for optimising intraoral scanners in CL/P are:

#### 4.3.1. Scanning Tip

The influence of scanning tip size on the accuracy of intraoral digital scans in patients with CL/P remains underexplored. Okazaki et al. and Abreu et al. reported difficulties in recording the deepest cleft areas due to limited access with standard tips [28,33]. In cases involving large oronasal fistulas, it was noted that obtaining precise three-dimensional data was particularly challenging unless the scanner tip was sufficiently small to enter and record the internal surface [57,58]. These findings highlight the need for redesigned tips that combine sufficient miniaturisation for neonatal use with maintained optical precision. A laboratory comparison of Carestream (standard and side-tip) and Trios 4 scanners found comparable accuracy for alveolar cleft depth (*p* > 0.05), yet none could consistently capture the deepest regions [38].

#### 4.3.2. Scanning Strategy

The rehabilitation of patients with a cleft lip and palate begins shortly after birth and continues through adolescence and adulthood [59]. Consequently, intraoral scanning in these patients spans across developmental stages: from edentulous arches in neonates, to partially dentate arches in infancy, and fully dentate arches in adulthood. While the accuracy of complete arch scanning in edentulous adults is comparable to CI [60]. Comparative studies of complete arch scanning of dentulous and edentulous arches reveal that edentulous arch scanning is particularly imprecise [61]. Since the scan of CL/P patients involves edentulous stages in neonates, partially dentulous stages in infants, and fully dentulous stages in later years, the accuracy of IOS can vary. Considering these factors, an optimal scanning strategy must be developed for CL/P patients to ensure accuracy.

Manufacturers’ recommended scanning strategies vary based on the technologies used, which can influence accuracy and quality. The scan path, the scanner’s position relative to the tissue surface, and maintaining a consistent distance during scanning can affect the quality of the scan [62,63]. However, limited evidence exists on optimal strategies for neonates with CL/P. In a laboratory study, two approaches were investigated: the traditional *cleft-unobstructed* technique and the *cleft-obstructed* method, as described by Weise et al., where the cleft region is temporarily filled with soft material to make the arch continuous. Findings revealed that cleft-obstructed scanning significantly reduced scan time and the number of scan interruptions across all scanners tested. Nevertheless, neither strategy successfully captured the deepest part of the alveolar cleft, suggesting that current techniques are inadequate for comprehensive imaging in neonates with CL/P [38]. These results indicate the need for further refinement of scanning strategies and clinician training to improve clinical outcomes.

#### 4.3.3. Scanner Type

The accuracy and clinical applicability of intraoral scanners (IOS) in full-arch and complex morphology scanning have been evaluated by several studies, including those by Amornvit et al., Michelinakis et al., and Kernen et al. [64,65,66]. Intraoral scanners are digital devices composed of a portable camera, computer, and processing software, used to capture three-dimensional representations of dental and soft tissue structures, typically stored in STL file format.

The scanning methodologies employed are diverse and rely on the applied technology. These include passive techniques, such as ambient light, and active techniques, such as structured light, encompassing triangulation, confocal imaging, Active Wavefront Sampling (AWS), and stereophotogrammetry. The quality and usability of the intraoral scanners are influenced by the different approaches used by the systems to capture images and calculate distances to the surface to be scanned [67]. Moreover, each scanner is equipped with unique technologies and sensors that directly affect the dimensions and weight of the scanning unit [68]. Hence, identifying the scanning technique that captures even the deepest part of the cleft while maintaining faster scanning is a prerequisite of an ideal scanner in patients with a CL/P.

In an investigation, three scanners—Trios 4, Carestream, and iTero—were assessed for scan time, interruptions, and scan quality using both cleft-obstructed and unobstructed strategies. Although no statistically significant differences were observed between devices in efficiency metrics, all scanners failed to capture the deepest portion of the cleft [60].

Collectively, these results reveal a current limitation of IOS technologies in managing CL/P cases and underscore the need for continued innovation. Scanner tip design, resolution, and adaptive software may all require modification to meet the demands of neonatal cleft imaging. Future research should focus on developing purpose-built systems or enhanced protocols specifically for use in this patient population.

This review provides a comprehensive synthesis of the current applications of digital technologies in cleft care, identifying existing gaps and practical challenges. Unlike previous reviews, it specifically examines variations in accuracy, clinical outcomes, and implementation feasibility across different digital systems. The insights generated highlight emerging opportunities for optimising digital workflows and guiding future research directions in this evolving field.

Future research should systematically evaluate scanning tip size, scanner type, and optimal scanning strategies to improve the accuracy and efficiency of DIs in cleft lip and palate (CL/P) management. Comparative studies of scanner technologies and protocols are needed to identify the most reliable approaches for capturing complex CL/P anatomy, particularly in neonates and infants. Well-designed controlled trials comparing digital and conventional methods, along with longitudinal studies on performance, safety, cost-effectiveness, and patient outcomes, are essential to establish evidence-based guidelines. Research should also explore integration with advanced digital workflows and operator training to standardise protocols and enhance clinical decision-making in CL/P care.

## 5. Study Limitation

This narrative review summarises the available clinical literature on the use of DIs (DI) in cleft lip and palate (CL/P). However, the methodological quality and risk of bias of the included studies were not formally assessed, and the limited number of high-quality investigations—many being small case series or pilot studies—may reduce the robustness of the conclusions. Few studies have systematically evaluated the challenges of intraoral scanning in neonates and infants or validated the reported advantages through controlled comparisons, and considerable variability in scanner types, scanning protocols, and outcome measures further hinders direct comparison. Additionally, this review was limited to studies published in English, which may have introduced publication bias and restricted the inclusion of relevant evidence from non-English sources.

## 6. Conclusions

This review highlights the growing role of DIs (IOS) in cleft lip and palate (CL/P) care. Current evidence suggests that IOS offers accuracy comparable to CI while improving patient comfort and reducing procedure time. Clinicians value IOS for its adaptability and lower risk of respiratory complications, particularly in newborns. DIs enhance diagnosis, treatment planning, and outcome evaluation. Future research should refine scanning strategies and algorithms to improve the accuracy and reliability of alveolar cleft recordings.

## Figures and Tables

**Table 1:** Search strategy used.

Database	Search Strategy
Pubmed	cleft*[Title/Abstract] AND (lip*[Title/Abstract] OR palate*[Title/Abstract] OR orofacial[Title/Abstract] OR alveolus[Title/Abstract])) AND ((intraoral[Title/Abstract] AND scan*[Title/Abstract]) OR “3D scan*”[Title/Abstract] OR “digital model*”[Title/Abstract] OR “digital impression*”[Title/Abstract] OR “3D model*”[Title/Abstract] OR “digital workflow*”[Title/Abstract] OR “digital work flow”[Title/Abstract] OR “3D print*”[Title/Abstract])
Scopus	TITLE-ABS-KEY ((“cleft lip and palate” OR “orofacial cleft*” OR “alveolar cleft”) AND (“intraoral scan*” OR “3D scan*” OR “digital impression*” OR “digital workflow*” OR “3D print*” OR “computer aided” OR “CAD”)) AND (LIMIT-TO (SUBJAREA, “MEDI”) OR LIMIT-TO (SUBJAREA, “DENT”)) AND (LIMIT-TO (LANGUAGE, “English”))
Web of Science	ALL = ((“cleft lip and palate” OR “orofacial cleft*” OR “alveolar cleft”) AND (“intraoral scan*” OR “3D scan*” OR “digital impression*” OR “digital workflow*” OR “3D print*” OR “computer aided”))
Cochrane Library	((“cleft lip and palate” OR “orofacial cleft*” OR “alveolar cleft”) AND (“intraoral scan*” OR “3D scan*” OR “digital impression*” OR “digital workflow*” OR “3D print*” OR “computer aided” OR “CAD”))
Embase	(cleft*[Title/Abstract] AND (lip*[Title/Abstract] OR palate*[Title/Abstract] OR orofacial[Title/Abstract] OR alveolus[Title/Abstract])) AND ((intraoral[Title/Abstract] AND scan*[Title/Abstract]) OR “3D scan*”[Title/Abstract] OR “digital model*”[Title/Abstract] OR “digital impression*”[Title/Abstract] OR “3D model*”[Title/Abstract] OR “digital workflow*”[Title/Abstract] OR “digital work flow”[Title/Abstract] OR “3D print*”[Title/Abstract])
Google Scholar	(“cleft lip and palate” OR “orofacial cleft*” OR “alveolar cleft”) AND (“intraoral scan*” OR “3D scan*” OR “digital impression*” OR “digital workflow*” OR “3D print*” OR “computer aided”)

**Table 2:** Overview of the 27 studies (2016–2024) on DIs and direct digital models in cleft lip and palate care, categorised by focus on accuracy, patient comfort, time efficiency, diagnostic use, treatment planning, and outcome assessment.

References		DI with Intraoral Scanner or Direct Digital Models (DDM) in the Care of Patients with CL/P					
	Accuracy	Effect of Scanning Parameters on Scanning Efficiency	Patient Comfort	Time Required	As Diagnostic Aids	Tx Planning/Treatment	As Outcome Measures
	N = 7	N = 1	N = 5	N = 5	N = 4	N = 10	N = 4
Chalmers, 2016 [15]	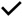		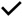	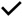			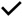
Choi, 2019 [16]					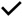		
Dalessandri, 2019 [7]	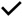		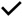			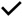	
Fomenko, 2019 [17]							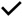
Ahmed, 2019 [18]						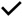	
Patel, 2019 [12]	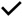			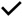	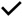		
Adrien Naveau, 2020 [19]						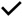	
Batra, 2020 [20]						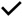	
Bous, 2020 [21]						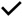	
Gong, 2020 [22]						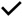	
Shanbhag, 2020 [23]						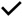	
Wang, 2021 [24]						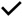	
Weise, 2021 [25]			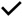	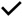			
Zeidan, 2021 [26]	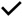						
Woodsend, 2022 [27]					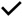		
Abreu, 2022 [28]			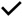				
Benitez, 2022 [29]			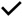	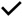			
Carter, 2022 [30]							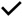
ElNaghy, 2022 [31]	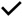			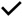			
Viñas, 2022 [32]							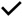
Zarean, 2022 [8]						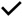	
Okazaki, 2023 [33]	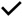						
Zhang, 2023 [34]					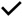		
Meyer, 2023 [35]						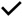	
Soliman, 2023 [36]	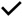						
Unnikrishnan, 2024 [37]	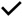						
Unnikrishnan, 2024 [38]		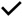					

**Table 3:** Accuracy of Intraoral Scanners.

Author and Year	Intervention	Scanner Used	Comparison	Outcome Measured	Population	Sample Size	Age	Result
Patel, 2019 [12]	Intraoral scanner	Trios 3 Shape	Indirect digital model (From Alginate impression)	Surface discrepancy between superimposed models.	Male; infant with BCL/P	1	3 months old	DI demonstrates comparable accuracy to CI
Chalmers, 2016 [15]	Intraoral scanner	Trios 3 Shape	Indirect digital model (From alginate impression)	GOSLON and modified Huddart Bodenham (MHB) indices	Non-syndromic UCL/P	43	Between 9 and 21 years	DI demonstrates comparable accuracy to CI
Dalessandri, 2019 [7]	Intraoral scanner	CS3600, Carestream Dental	Indirect digital model (From tray and Putty)	Difference in the outcome of or by Intra-arch measurements	UCL/P and BCL/P	6	Newborn	DI demonstrates comparable accuracy to CI
Zeidan and Kamiloglu, 2021 [26]	Intraoral scanner	CEREC Omnicam	CBCT	Intra-arch measurements	Plaster models of both sexes CL/P	44	Models of infants up to 6 months of age.	DI demonstrates comparable accuracy to CI
Elnaghy, 2022 [31]	Intraoral scanner	Trios 3 Shape	Indirect digital model (From alginate Impression)	3-D surface model discrepancy by superimposition	Male and female UCL/P	2	4-week-old girl 5–week–old boy	DI demonstrates comparable accuracy to CI
Okazaki, 2023 [33]	Intraoral scanner	Trios 3 Shape	Indirect digital models	Intra-arch measurements 3-D surface model discrepancy by superimposition	Male and female UCL/P	7	Mean age of 108 days	DI demonstrates comparable accuracy to CI
Soliman, 2023 [36]	Intraoral scanner	Medit i700, Medit Corp., Seoul, Republic of Korea	Indirect digital model (From alginate Impression)	Intra-arch measurements 3-D surface model discrepancy by superimposition	Male and female UCL/P	7	infants	DI demonstrates comparable accuracy to CI
Unnikrishnan, 2024 [37]	Intraoral scanner	Trios 3 Shape	Indirect digital models (From rubber-based &alginate Impression)	Intra-arch measurements 3-D surface model discrepancy by superimposition	Soft acrylic models	42	Neoantes	DI demonstrates comparable accuracy to CI

**Table 4:** Clinician and patient/parents reported outcome.

Author and Year	Objective	Population	Sample Size	Age	Method of Assessment	Clinician-Reported Outcome	Patient/Parent Related Outcome
Chalmers, 2016 [15]	To evaluate intraoral 3D scans for assessing dental arch relationships and obtain patient/parent perceptions of impressions and intraoral 3D scanning.	Non-syndromic unilateral cleft lip and palate	43	5–21 years	Questionnaire	-	Patients had higher ratings for scanning comfort than impressions and for scanning time than impressions.
Dalessandri, 2019 [7]	To evaluate the accuracy, invasiveness and impact on clinical results of a digital oral impression protocol in the PSOT of newborn cleft lip and palate (CL/P) patients undergoing primary alveolar surgical repair.	BCL/P and UCL/P	6	Newborn	Questionnaire	Repetition of Impressions: The scanner head, preheated, facilitated scanning, resulting in approximately 30 s of scanning time, with no repetition of DI compared to CI. Clinician’s experience: The clinician who took all the impressions considered the IOS method to be less stressful compared to the T&P method.	The scanner head, preheated, facilitated scanning, resulting in approximately 30 s of scanning time, with no repetition of DI compared to CI. Parents of children preferred DI
Patel, 2019 [12]	To document the innovative use of a digital impression technique to assess arch form in an infant with bilateral CL/P.	Male; infant with BCL/P	1	3 months old	Observation	Time required:1 min	
Weise, 2021 [25]	To evaluate intraoral scanning (IOS) in infants, neonates, and small children with craniofacial anomalies for its feasibility, scanning duration, and success rate.	Neonates, infants and small children with craniofacial disorders, including CL/P	141	Median age of 137 days.	Observation	Median scanning duration-151s (36–537) in CL/P patients Longest scanning duration in CL/P patients IOS in 4 CL/P patients was repeated. One CL/P patient, IOS could not be done	
Benitez, 2022 [29]	To investigate the implementation and risks of digital impressions for the youngest patients with orofacial clefts.	Children with CL/P	342	Median age of 8.7 months.	Observation	No adverse device events or adverse events No repetition of scans Median scan duration of 85.5 in cleft palate 50 s for cleft lip and nose scan Younger patients need more time for intraoral scanning. No significant difference in scanning time between awake and anesthetized patients (*p*-value > 0.05) Cleft type affects scanning duration in awake patients	
Abreu, 2022 [28]	To show the clinical use of an intraoral digital impression in the fabrication of obturator/speech aid appliances in children with cleft lip and palate deformity.	Children with repaired bilateral cleft lip and palate and isolated cleft of the hard and soft palate	2	4–5 years	observation	Trimming the digital casts is challenging due to the depth of the cleft and the software’s struggle in recognising the most apical portion of the cleft deformity	-
ElNaghy, 2022 [31]	To evaluate the accuracy of intraoral digital impression compared to conventional impression in patients with CL/P	Male and female UCL/P	2	4-week-old girl 5–week–old boy	observation	Time required: 80–120 s	

**Table 5:** Effectiveness of Intraoral scanners/Direct digital models in the clinical care of patients with CL/P.

Author &Year	Purpose	Population	Age	Sample Size	Criteria Assessed
Choi, 2019 [16]	Diagnostic	children with CL/P	Mean age of 13 months	3	Maxillary arch dimension and cleft size
Patel, 2019 [12]	Diagnostic	Infants with BCL/P	3 months	1	Arch form
Woodsend, 2021 [27]	Diagnostic	239 models, of which 161 are from cleft palate	5 years	239	Identification of landmarks and the modified Huddart-Bodenham scoring system.
Zhang, 2023 [34]	Diagnostic	Patients with UCL/P& BCL/P	-	18	Stable areas of the maxillary arch
Dalessandri 2019 [7]	Treatment planning, Treatment, outcome measures	infants with CL/P	Newborn	6	Fabrication of using nasoalveolar moulding plate using digital models. Treatment changes with digital protocol and conventional nasoalveolar moulding.
Ahmed, 2019 [18]	Treatment planning,	------	------	-----	Construction of NAM plate
Shanbhag, 2020 [23]	Treatment planning & Treatment,	Infant with CL/P	2 months	1	Construction of NAM plate
Naveau, 2020 [19]	Treatment planning,	Newborn with Unilateral CL/P 4-year-old girl with primary cleft lip repair	3 weeks	2	Construction of NAM plate
Gong, 2020 [22]	Treatment planning Treatment & Outcome measures	Infants with CBCL/P	Mean age of 1.1 weeks	9	Fabrication of CAD-NAM Comparison of pre- and post-treatment
Bous, 2020 [21]	Treatment planning & Treatment	Infants with CUCL/P	21 Days	1	Fabrication of 3D printed clear aligner NAM device.
Batra, 2020 [20]	Treatment planning & Treatment	Infants with UCL/P	1 month	4	Fabrication of 3D printed clear aligner NAM device.
Wang, 2021 [24]	Treatment planning & Treatment	Pierre Robin syndrome with CL/P	7 years	1	Fabrication of a custom-fitted temporary vacuum-formed prosthetic obturator
Zarean, 2022 [8]	Treatment planning & Treatment	Newborn with CL/P	newborn		Fabrication of CAD-NAM
Chalmers, 2016 [15]	Outcome measures	Patients with UCL/P	Between 5 and 21 years	43	GOSLON and MHB indices to evaluate the dental arch relationship as a measure of a surrogate for primary surgery outcome.
Carter, 2022 [30]	Treatment planning Treatment & Outcome measures	Infant with UCL/P	4.5 months	1	Extra-oral facial scans and intra-oral impressions are compared between 3 timepoints: pre-treatment, post-treatment with NAM, and postsurgical treatment.
Viñas, 2022 [32]	Outcome Measures	CL.UCL/P, BCL/P, CPO	Young Adults	83	Craniofacial growth alterations
Fomenko, 2019 [17]	Outcome measure	Children with CBCL/P	3–4 years	22	Premaxilla’s size and position

## Data Availability

No new data were created or analyzed in this study.
